# Persistence of High In Vivo Efficacy and Safety of Artesunate–Amodiaquine and Artemether–Lumefantrine as the First- and Second-Line Treatments for Uncomplicated *Plasmodium falciparum* Malaria 10 Years After Their Implementation in Gabon

**DOI:** 10.2478/s11686-019-00115-y

**Published:** 2019-09-11

**Authors:** Jacques M. Ndong Ngomo, Guy J. Ondzagha Megnie, Bridy Moutombi Ditombi, Jeanne V. Koumba Lengongo, Noé P. M’Bondoukwé, Christelle L. Offouga, Denise P. Mawili-Mboumba, Jean B. Lekana-Douki, Pascal Ringwald, Thierry Fandeur, Marielle K. Bouyou-Akotet

**Affiliations:** 1 Department of Parasitology and Mycology, Faculty of Medicine, University des Sciences de la Santé, BP 4009, Libreville, Gabon; 2 Unité de Parasitologie Médicale (UPARAM) CIRMF, Franceville, Gabon; 3 Global Malaria Program, World Health Organization, 1211 Geneva, Switzerland

**Keywords:** *Plasmodium falciparum*, Therapeutic efficacy, Safety, ACT, Gabon

## Abstract

**Purpose:**

Artesunate–amodiaquine (AS–AQ) and artemether–lumefantrine (AL) have been widely used for the treatment of uncomplicated *Plasmodium falciparum* malaria since 2005 in Gabon. Since 2011, a rebound of malaria morbidity has been observed in this country, while no survey evaluating ACT efficacy was performed. During the same period, parasite resistance against artemisinin has been reported in Asia. The aim of this study was to assess the efficacy and tolerability of these two drugs in two sentinel sites of Gabon 10 years after their implementation.

**Methods:**

Children aged from 12 to 144 months with uncomplicated malaria were recruited at the Regional Hospital of Melen, Libreville and in the Urban Health Center of Franceville between March 2014 and September 2015. The therapeutic efficacy was evaluated according to the WHO 2008 protocol of 28-day follow-up and PCR-uncorrected/corrected treatment outcomes were assessed.

**Results:**

One hundred and eighty-five children (98 ASAQ and 89 AL) were followed up until day 28. The PCR-corrected ACPR was 98.9% for AS–AQ and 96.4% for AL. Late therapeutic failure rate was 3.6% and 1.1% for AL and AS–AQ, respectively (*p* = 0.2). Adverse events and serious adverse events were rarely observed with both treatments.

**Conclusion:**

AS–AQ and AL are still efficacious and well-tolerated for the treatment of uncomplicated malaria in Gabonese children.

## Introduction

Since 2000, the majority of malaria-endemic countries substituted monotherapies by artemisinin-based combination therapies (ACTs) for uncomplicated malaria treatment [20]. Following ACTs use, a reduction of malaria mortality by 62% globally between 2000 and 2015, and by 29% between 2010 and 2015 was estimated [23]. Resistance to artemisinin and its spread are now reported in South-East Asia. This region is known to have been focus of origin of antimalarial drug resistance before it reaches Africa [2, 6].

In Gabon, malaria is the second leading cause of hospitalization in pediatrics wards after respiratory tract infections; it is responsible for more than one-third of all febrile patients [4]. Almost all cases are due to *P. falciparum*, of which strains with molecular markers of artemisinin partner drugs are highly frequent in different areas of the country [11, 13].

At the time of artesunate–amodiaquine (AS–AQ) and artemether–lumefantrine (AL) implementation in public health center as the first- and second-line treatments for uncomplicated malaria (2003–2005), respectively, their efficacy was estimated at 99–100% [1, 3]. However, reports from sentinel sites for malaria surveillance highlighted a reduced AS–AQ prescription in health centers, frequent stock outs of AL and an increase of malaria prevalence, 7–10 years later [14]. In 2013, the national recommendations for uncomplicated malaria treatment changed and both drugs were adopted as first-line treatments, while dihydroartemisinin–piperaquine (DHAP) was selected as the second-line treatment [14].

This study presents the results of a clinical trial evaluating AS–AQ and AL efficacy and tolerability in two cities of Gabon (Libreville and Franceville), where both molecular markers of resistance as well as a rebound of malaria morbidity have been previously described [8, 12].

## Materials and Methods

### Study Sites and Procedure of Patients’ Screening

This prospective study was carried out in two sentinel sites for malaria surveillance between March 2014 and September 2015 in Gabon. Precisely, the study sites were the Operational and Clinical Research Unit (OCRU) of the Regional Hospital of Melen (RHM), which is located at 11 km from Libreville (West part of Gabon) and the Urban Health Center of Franceville (south-east region of the country). In both cities, malaria transmission is perennial, with an entomological inoculation rate estimated at 33.9 infected bites per person per year [15]. *P. falciparum* represents more than 93% of the parasite species diagnosed [12, 15].

During the study period, children aged from 12 to 144 months with the following criteria were approached: febrile (tympanic temperature of > 37.5 °C) or with a 24–48 h history of fever before the day of consultation, ability to swallow oral medication, willingness to comply with the duration of the study, no signs of complicated malaria as per WHO guidelines [21], ability to attend the outpatient clinic on stipulated days for the follow-up, and parent or guardian written informed consent for the child participation in the study. Children were first screened for malaria using the SD Bioline Pf/pan rapid diagnosis test RDT [5]. In case of RDTs’ positivity, thick and thin blood smears were performed to determine the parasite density and to identify the species [16]. Each child was included if he/she had a *P. falciparum* mono infection with a parasite count between 2000 and 200,000 asexual forms per microliter of blood (p/µL). Criteria of exclusion were the presence of complicated malaria as defined by WHO 2000 [19], a mixed *Plasmodium* infection, concomitant disease, or other danger signs, known allergy to AS–AQ and AL, presence of a treatment with an antimalarial drug in the previous 3 weeks.

### Treatment

After enrollment, participants received appropriate dose of AL (Coartem^®^ Novartis) and AS–AQ (Coarsucam^®^, Sanofi Aventis) at the study site during three consecutive days. They were observed within 1 h after administration. Children who vomited less than 30 min after drug administration were given a second dose and were observed for 30 min more. They were monitored according to the 28-day-WHO protocol (WHO 2003).

For each patient, the outcome of the treatment was classified based on clinical and parasitological tests as early treatment failure (ETF), late treatment failure (LTF), late clinical failure (LCF), late parasitologic failure (LPF), and adequate clinical and parasitological response (ACPR) as per WHO, 2003 protocol [22].

In case of therapeutic failure, patients were retreated with dihydroartemisinin–piperaquine phosphate combination (DHAP). Recrudescence and reinfection cases were distinguished using merozoite surface protein 1 (block 2) and 2 (block 3) gene genotyping by PCR as previously described [18].

### Statistical Analysis

All data were entered into an Excel-based program and analyzed using the computer program developed by WHO for antimalarial drug efficacy. This software includes formula for the classification of treatment outcome with and without PCR. Patients who were excluded or withdrew from the study were not included in the per protocol analysis.

### Ethical Consideration

The study was conducted in accordance with the principles of good clinical practices (GCP) and reviewed and approved by Gabonese National Ethics Committee and the World Health Organization (WHO) Ethical Review Committee. A written informed consent was obtained from parents or guardians of all study participants.

## Results

### Study Population Characteristics

**Fig. 1:**
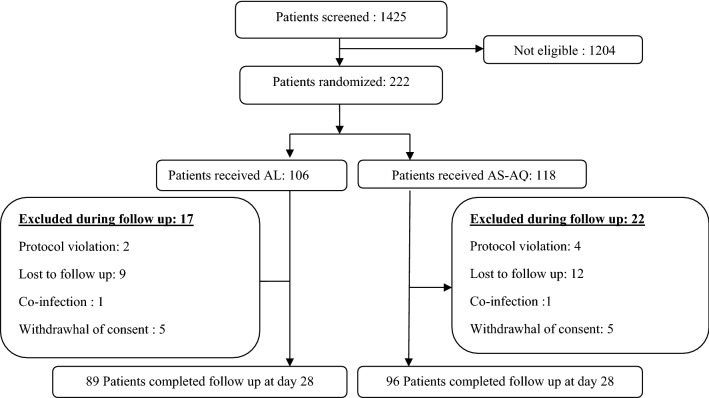
Patients’ screening profile

A total of 222 of the 1425 screened children fulfilled the inclusion criteria. Among them, 116 patients received AS–AQ and 106 AL. The excluded patients during the follow-up represented 17.3% (*n* = 39/225): 17 from the AL group vs 22 from AS–AQ. The reasons for exclusion are shown in the trial profile (Fig. 1). The mean temperature [34.4 °C (± 1.2) vs 38.5 °C (± 1.3)] and the median density parasite (20,542 [1715–179,000] p/µL vs 27,941 [1127–198,800] p/µL) were significantly higher in the AS–AQ group compared to AL, respectively (*p* = 0.04).

### Treatment Outcome

In both groups, the parasite clearance was obtained from the second day following the first dose administration. The results of the treatment efficacy before and after PCR correction are reported in Table 1. After PCR correction, the frequency of ACPR was similar between AS–AQ (98.9%, *n* = 89) and AL (96.4%, *n* = 82) treatment groups (*p* = 0.3).

**Table 1:** Treatment outcome of artesunate–amodiaquine (AS–AQ) and artemether–lumefantrine (AL)

Outcome	AL	AS–QA	*p* value
PCR-uncorrected day 28 cure rates			
Per protocol, *n*	89	96	
ACPR, *n* (%)	82 (92.1)	89 (92.7)	0.55
LPF, *n* (%)	5(5.6)	6 (6.3)	
LCF, *n* (%)	2 (2.2)	1 (1.0)	
Cumulative failure, *n* (%)	7 (8.5)	7 (7.8)	
PCR-corrected day 28 cure rates			
Per protocol, *n*	85	90	
ACPR, *n* (%)	82 (96.4)	89 (98.9)	0.3
LPF (recrudescence), *n* (%)	2 (2.4)	1 (1.1)	
LCF (recrudescence), *n* (%)	1 (1.2)	0 (0)	
Cumulative failure	3(3.6)	1 (1.1)	

Patients with re-infections and individuals for whom the outcome could not be assessed by PCR were censored from the PCR-correction analysis

The microscopic gametocytemia was cleared from day 1 for AL and day 3 for AS–AQ-treated children.

### Tolerability and Safety

**Fig. 2:**
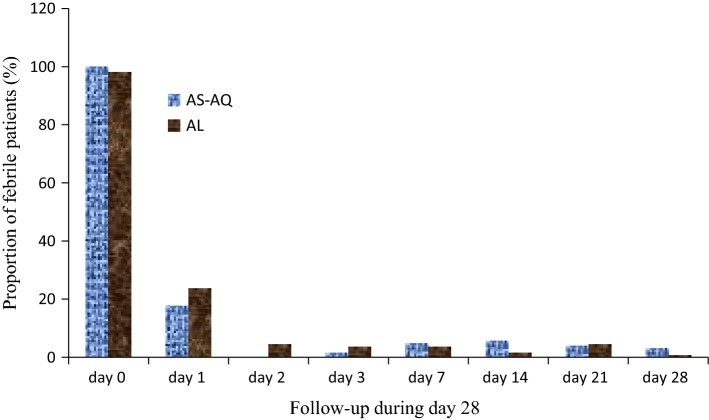
Fever clearance in the two groups of treatment

All the recruited patients recovered rapidly during the first 72 h of follow-up (Fig. 2). Minor and serious adverse events were rarely observed in AS–AQ vs AL groups, respectively: vomiting 2.0% (*n* = 2) vs 5.6% (*n* = 5) (*p* = 0.2), cough 16.6% (*n* = 16) vs 6.7% (*n* = 6) (*p* = 0.004), asthenia 14.5% (*n* = 14) vs 5.6% (*n* = 5) (*p* = 0.05), and abdominal pain 2.0% (*n* = 2) vs 2.2 (*n* = 2) (*p* = 0.6). Hemoglobinuria (*n* = 1) and convulsion (*n* = 1) were found in only one patient treated with AS–AQ and in one child who received AL.

## Discussion

10 years after the implementation of AS–AQ and AL for the treatment of uncomplicated malaria in Gabon, it was necessary to re-evaluate the therapeutic efficacy of these combinations. This clinical trial was performed in two sentinel sites for malaria surveillance according to the WHO procedures. The data obtained confirmed that both drugs remain efficacious and well-tolerated in Gabon. Temperature recovery from day 0 to day 28 occurred swiftly in both treated groups as reported by other authors through assessment of these two drugs. Likewise, the occurrence of other adverse events was rare [17, 24].

PCR-corrected cure rates for AL and AS–AQ were similar (96.5%) and remain high and adequate according to WHO recommendations. Others studies reported cure rate varying from 99.3 to 100% after treatment with AS–AQ and from 99.3 to 100% for AL [7, 24]. In the present study, the efficacy rates were obtained when ACTs coverage was below 60% in the country. This clinical trial is the first performed in Franceville, where the frequency of molecular markers associated with the resistance of *P. falciparum* to AQ and Lumefantrine is non negligible [9]. The relatively and non-significant lower efficacy of AL, in comparison with AS–AQ and also to the previous historical estimates, could be a first signal of a potential decline of AL parasite effect. However, fever and microscopic gametocytemia clearance tended to be longer among patients treated with AS–AQ, compared to AL. The efficacy of the AS–AQ combination on gametocytes clearance has been reported by other authors [10]. Both ACTs appeared to be safe and well-tolerated, although vomiting events were associated with the use of AL in five patients.

AL and AS–AQ are still efficacious and well-tolerated in Gabon. Their use as first-line treatment is not compromised. Continuous monitoring throughout the country is necessary to allow early detection of decline efficacy of both drugs.
